# Detection and Drug Susceptibility Testing of *Neisseria gonorrhoeae* Using Isothermal Microcalorimetry

**DOI:** 10.3390/microorganisms9112337

**Published:** 2021-11-11

**Authors:** Anabel E. Grütter, Tecla Lafranca, Aurelia Pahnita Sigg, Max Mariotti, Gernot Bonkat, Olivier Braissant

**Affiliations:** 1 Department of Biomedical Engineering, University of Basel, Gewerbestrasse 14, 4123 Allschwil, Switzerland; anabel.gruetter@stud.unibas.ch (A.E.G.); tecla.lafranca@stud.unibas.ch (T.L.); aureliapahnita.sigg@stud.unibas.ch (A.P.S.); max.mariotti@stud.unibas.ch (M.M.); 2 alta uro AG, Centralbahnplatz 6, 4051 Basel, Switzerland; Bonkat@alta-uro.com

**Keywords:** isothermal microcalorimetry, *Neisseria gonorrhoea*, antimicrobials

## Abstract

Background: Gonorrhea is a frequently encountered sexually transmitted disease that results in urethritis and can further lead to pelvic inflammatory disease, infertility, and possibly disseminated gonococcal infections. Thus, it must be diagnosed promptly and accurately. In addition, drug susceptibility testing should be performed rapidly as well. Unfortunately, *Neisseria gonorrhoea* is a fastidious microorganism that is difficult to grow and requires culturing in an opaque medium. Methods: Here, we used isothermal microcalorimetry (IMC) to monitor the growth and the antimicrobial susceptibility of *N. gonorrhoea*. Results: Using IMC, concentrations of *N.* *gonorrhoea* between 2000 and 1 CFU·mL^−1^ were detected within 12 to 33 h. In addition, drug susceptibility could be monitored easily. Conclusions: The use of isothermal microcalorimetry provides an interesting and useful tool to detect and characterize fastidious microbes such as *N. gonorrhoea* that require media incompatible with optical detection conventionally used in many commercial systems.

## 1. Introduction

Gonorrhea is the second most common bacterial sexually transmitted disease (STI) worldwide. According to the World Health Organization (WHO), there were around 87 million new infections in 2016 [1,2]. The causative pathogen of Gonorrhea, *Neisseria gonorrhoea* (Ng), is a Gram-negative diplococcus that infects the urogenital tract as well as the rectum and the pharynx [3,4,5]. Men with such a urogenital infection are often symptomatic, presented as urethritis [3,4]. On the contrary, most women are asymptomatic. Symptoms (if any) include urethritis or cervicitis and are often non-specific. Rectal and pharyngeal Gonorrhea are present in both genders and remain mostly asymptomatic [4,6,7]. Due often non-specific symptoms or even asymptomatic disease progressions, Gonorrhea can extend into an ascending infection. Complications include pelvic inflammatory disease (PID), ectopic pregnancy, infertility and possibly disseminated gonococcal infections (DGIs) [8,9]. Unfortunately, because many patients remain asymptomatic and the increase in sexually risky behavior, the incidence continues to rise.

Furthermore, the time and effort required for the detection of *N. gonorrhoea* and the determination of its antimicrobial susceptibility are high. For these reasons, an empirical antimicrobial therapy is often chosen as first-line treatment. This treatment consists of a single dose of ceftriaxone in combination with azithromycin (see details in EAU guidelines [10]). As with other bacteria, *N. gonorrhoea* is also able to develop resistance to various antibiotics, including the first-line ones and many others [11]. Due to the emergence of resistance and the consequent ineffectiveness of the antibiotics used, antibacterial susceptibility testing becomes crucial to ensure effective and targeted therapy. Minimizing inadequate treatment also reduces the emergence of new resistance [12]. Because of the growing number of cases, the inefficiency of the currently applied diagnostic methods and the emergence of new resistant strains, diagnostic methods that allow faster and targeted treatment are urgently needed.

Currently, diagnosis of *N. gonorrhoea* relies mostly on a urethral swab followed by microscopy, microbial culture or nucleic acid amplification test (NAAT) such as PCR or LAMP. In high income settings the nucleic acid amplification test (NAAT) is the most used test to diagnose Gonorrhea, whereas it is rarely used in low-income settings, due to the high associated costs [13,14,15]. NAAT has a high specificity and sensitivity both in urethral gonococcal infections and extragenital gonococcal infections. However, it is recommended that a positive test result from an extragenital infection should be confirmed with an alternative method due to the possibility of cross reactivity with commensal or atypical *Neisseria* species [16,17,18,19]. In addition, NAAT may be superior to culture in some respects, for example in sensitivity, ease, and speed of performance, as shown in many studies. However, it lacks the possibility of direct antimicrobial susceptibility testing [20,21,22]. Therefore, there is still the need for a new method, which can both detect *Neisseria* and allows determination of antimicrobial resistance pattern in an easy and practical manner.

In this context, we investigated the use of isothermal microcalorimetry (IMC) for the detection and antimicrobial susceptibility testing of *N. gonorrhoea.* For microbiologists, *N. gonorrhoea* is considered to be a fastidious microorganism that requires specific growth medium (such as chocolate agar, GC medium) that are solid and/or opaque due to the presence of components such as agar (to make the medium solid), starch and hemoglobin (making the medium cloudy or even opaque). Isothermal microcalorimetry measures metabolic heat produced by active or replicating microorganisms in a given medium [23,24]. Isothermal microcalorimetry (IMC) is a laboratory method for the measurement and recording of real-time heat production rate (heatflow) at microwatt levels (μJ/sec = μW) during bacterial growth (i.e., heatflow curve) [25,26,27,28]. As only heat is measured, IMC does not require a transparent or liquid medium, making it suitable for the detection of *N. gonorrhoea* in the medium. Previous studies have already shown promising results in this field. For example, in 2012 a study using IMC demonstrated that this method allows rapid detection of bacteriuria (in just 3.1 h), enables the pathogen to be targeted through different heat flow patterns and that low colony counts (10^3^ CFU/mL) are needed for the detection. Bacterial strains of the most common urinary tract pathogens were used in this study: *Staphylococcus aureus*, *Enterococcus faecalis*, *Proteus mirabilis* and *Escherichia coli* [28]. Braissant and colleagues demonstrated that IMC could be used to find an adequate antimicrobial therapy for urosepsis in just seven hours [29]. Regarding the drug susceptibility, isothermal microcalorimetry has already been used for various species such as MRSA [30], *Aspergillus* [31], *Mycobacterium* [32] and *S. aureus* and *E. coli* [33]. Finally, the use of solid and opaque medium has already been shown to be possible with IMC, especially in the context of tuberculosis diagnosis and drug susceptibility testing [34,35]. Given the reliability of these results, the aim of this study was to use this procedure in a field that to our knowledge has not yet been explored, namely antimicrobial susceptibility testing for *N. gonorrhoea* using IMC. Our aim was, therefore, to apply IMC to a bacterium that has not attracted much attention until now and to provide a sensitive, rapid and effective method of diagnosis and drug susceptibility testing that can ideally act as a means of point-of-care testing (POCT) in the clinical world.

## 2. Materials and Methods

### 2.1. Organisms

The strains of *N. gonorrhoea* (ATCC 19424 and ATCC 43069) were obtained as dehydrated pellets (Microbiologics, St Cloud, MN, USA) and stored at 4 °C before use. The pellets were rehydrated in sterile brain–heart infusion (BHI) medium previously autoclaved at 121 °C for 15 min according to the manufacturer’s instructions. The pellets were rehydrated 1 h before use. Pellets where homogenized using a grinding tool and through repeated vortexing. Following homogenization, the solution was used as inoculum for the different experiments described below. In addition, the inoculum bacterial concentration was determined by plating on Columbia agar with added 5% sheep blood (Becton Dickinson (BD), Franklin Lakes, NJ, USA) after serial 10-fold dilutions. An additional plate was prepared by streaking 10 µL of the undiluted inoculum on the same medium. All plates were incubated 48 h at 37 °C and visually inspected or counted after this time.

### 2.2. Detection in Various Medium

To test the effects of different supplement addition on the growth and thus further detection in urine or medium, supplements were added to the urine or the GC base medium (Condalab, Madrid, Spain) according to Table 1. GC medium base and hemoglobin solution were autoclaved separately at 121 °C for 15 min and mixed after cooling down. All other components were obtained sterile and added aseptically to obtain the final medium. Urine was obtained from voluntary healthy donors and was rapidly centrifuged to remove sediments (if any). A final filtration through a 0.2 µm filter (stericup) was performed to ensure sterility and clarity. After filtration, the sterile urine was used directly or stored at 4 °C until use if required. All additions to urine were made aseptically.

In addition, using the medium providing the best growth (i.e., ATCC 814 medium—see results section for details) we also performed a dilution series to determine the detection speed. Detection was considered positive when the signal passed above a threshold of 10 µW.

### 2.3. Drug Susceptibility Testing

Measurements performed in the previous section showed that GC medium (ATCC medium no. 814) containing hemoglobin (1% *w/v*) and Isovitale X (1% *v/v*) was the most effective medium for the cultivation of the *N. gonorrhoea* strains used (see results section for details). We therefore determined the drug susceptibility in this medium. For this proof of principle, we used ceftriaxone at the following concentrations: 4.17, 1.25, 0.42, 0.13 and 0.04 µg∙L^−1^. Sterile controls and controls without antimicrobials were subjected to the same conditions.

### 2.4. Calorimetry Procedure

All the samples were prepared in sterile 4 mL calorimetric glass ampoules. Then, 3 mL of sample was placed in the ampoule, and the ampoule was sealed. The ampoule was introduced into the calorimeter (TAM III, Waters/TA, New Castle, DE, USA) following the two-step thermal equilibration procedure recommended by the manufacturer. After 1 h of thermal equilibration, heat production rates (heatflow) were recorded until they returned to baseline or for at least 96 h. At the end of the experiment, data were resampled to obtain an effective sampling rate of 1 data point every 5 min and exported as a CSV file.

### 2.5. Statistical Analysis

All the calculations were performed in the statistics program R. The raw data (i.e., heatflow curves) were integrated to obtain heat over time curves. All further curve fitting was performed under the assumption that the heat produced during growth is proportional to the growth curve (i.e., that the cost of producing a bacterial cell remains constant) [36,37], and using the Gompertz growth model the maximal growth rate (µ), the lag phase (λ), and the maximum heat (Q_max_) were calculated from the heat over time curve [37,38,39]. Additionally, heatflow above 10µW was considered to be the threshold for positive detection, and the corresponding time was recorded.

## 3. Results

### 3.1. Effect of Medium and Supplements

**Figure 1:**
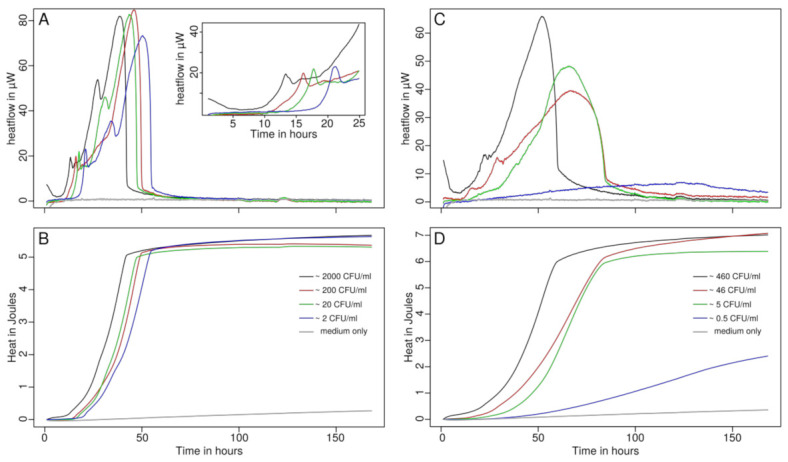
Growth of *N. gonorrhoea* ATCC 19424 (**A**,**B**) and ATCC 43069 (**C**,**D**) monitored using IMC. A: Heatflow of *N. gonorrhoea* ATCC 19424 and insert showing details of the initial 25 h. B: Heat produced by of *N. gonorrhoea* ATCC 19424 over time showing a curve similar to a growth curve. C: Heatflow of *N. gonorrhoea* ATCC 19424. D: Heat produced by of *N. gonorrhoea* ATCC 19424 over time showing a curve similar to a growth curve.

*N. gonorrhoea* ATCC 19424 was used to test for different growth in the different media. The results are summarized in Table 2. This strain of *N. gonorrhoea* was not cultivable in any urine-based medium. We assume that the concentration of necessary nutrients is too low even with morning urine. This means the detection of *N. gonorrhoea* with IMC in urine or artificial urine is compromised. However, using GC-based medium showed growth in all conditions. The growth was improved by the addition of Isovitale X supplement and hemoglobin to a lesser extent. The use of sheep blood did not improve growth and even showed a decrease (however, the decrease was not significant). Out of the used mediums and additions, the conventional growth medium (GC medium, ATCC medium no. 814) with hemoglobin and Isovitale X supplement performed the best since it showed the highest growth rate with a low lag phase compared to the other combinations. In this medium the growth pattern shows a main peak with additional smaller peaks that are found before the maximum activity is reached (see Figure 1). A possible explanation for the multiple peaks could be the use of different nutrients or terminal electron acceptors by *N. gonorrhoea* during its growth as previously described [24,27,40].

### 3.2. Time to Detection

To determine the time to detection, we used a 10 µW threshold corresponding to ca. 3.3·10^5^ CFU·mL^−1^ (assuming a 2 pW per cell heat production rate [41,42]). The serial dilution with the two selected strains of *N. gonorrhoea* led to measured detection times between 12 and 33 h depending on the strain and bacterial concentration used (that were of rather low concentration but comparable to those found in patient urine- Table 3). Only one sample with very low CFU counts of the ATCC 43069 strain did not pass the threshold of 10 µW (although it came close). This emphasizes that the differences between the strains of the same organism should be taken into account. In addition, we assume that it is likely that after sedimentation of the medium, only a micro-colony could develop, being trapped in the sediment. Increasing growth would have probably required stirring or shaking that is not possible with the used instrument.

### 3.3. Drug Susceptibility Testing

**Figure 2:**
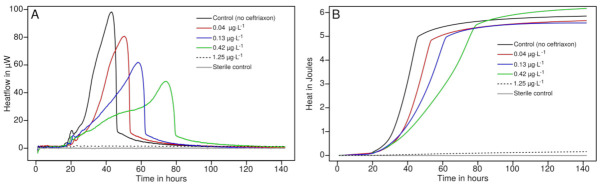
Heatflow (**A**) and heat over time (**B**) during the growth of *N. gonorrhoea* ATCC 43069 exposed to increasing concentrations of ceftriaxone.

When increasing concentrations of ceftriaxone were added up to 3× MIC, inhibition of *Neisseria* growth was clearly visible (Figure 2). The growth rate decreased from 0.29 to 0.00 h^−1^ at MIC, as the lag phase duration remained roughly similar for all the samples between 29.9 ± 1.5 and 36.1 ± 1.5. The MIC measured for both strains is consistent with literature values for these well characterized susceptible strains. Please note that at MIC level the strain ATCC 19424 shows considerably delayed growth and was clearly visible (although very slow) only after 100 h (data not shown). This shows that the methodology previously developed for urosepsis using a single concentration of antimicrobial to rapidly determine if a strain is susceptible or resistant is also applicable with *N. gonorrhoea*. However, due to the slow growth and fastidious nature of *Neisseria* species, it is likely that at least 12 to 15 h could be needed for such a discrimination.

## 4. Discussion

Overall, the use of the IMC is easier than culturing, and there is little preparation time. There is also the possibility of measuring antimicrobial susceptibility and rapidly discriminating between susceptible and resistant strains. Once an optimal medium has been chosen, detection is rather simple. In the development of calorimetric methods for the detection of this fastidious pathogens, other media such as Thayer–Martin agar or modified chocolate agar could be of interest [43]. However, in this study we focused on the use of liquid media as they are more suited for a practical use considering diagnostic applications and calorimetry. Still, the above-mentioned agar media could be of interest in the study of the formation of biofilm as these structures have been shown to form on epithelial cells and cervical cells during *Neisseria* infections [44]. In addition, future studies should also include the use of selective antimicrobial supplements for the direct isolation of *Neisseria* species [43].

The detection of *N. gonorrhoea* using isothermal microcalorimetry is slower than with NAAT, but it allows more evaluations once the IMC measurements are completed. Indeed, since the sealed ampoule still contains almost undisturbed sample with higher amounts of *Neisseria*, those bacteria can be recovered for additional identification or confirmation using MALDI-TOF or serological testing, for example. This is not possible with many NAAT-based techniques at this point but is still required as NAAT can be sensitive to other non-pathogenic but closely related *Neisseria* species [16,17,19].

In addition, we must emphasize that this study is very preliminary work and that many improvements are possible when considering the nature and composition of the medium. Indeed, as with non-motile microbial cells, hemoglobin (provided as dried red blood cells) and starch (insoluble) tend to sediment and are found at the bottom of the vial after the experiment. Therefore, it is very likely that the conditions for the growth of *N. gonorrhoea* are not homogenous throughout the vial and thus might not be optimal. Previous studies have shown that the use of Percoll or Ficoll that increase the density of the medium is a good solution to avoid such sedimentation, thus providing much more homogenous conditions for growth [45]. This; however, was not in the scope of the present study but will be considered and further investigated using similar media. There are also many other supplements such as Vitox or Yeast autolysate that could be used to make the growth of *N. gonorrhoea* and other *Neisseria* species faster. This is indeed of interest as IMC has been used for a long time to optimize growth condition for industrial purposes but also for human cell research [46,47,48]. Finally, we did not investigate selective supplements for direct determination and isolation from urine samples or swabs. This will also become of interest as the technique progresses.

With respect to antimicrobial susceptibility testing, our results show that with rather low concentration susceptibility could be determined within 12 h. At this point isolation would be needed before running a calorimetry measurement. As isolation takes 24 h and testing takes another 12 h, results can be expected within 36 h. Overall this is still 12 h faster than conventional methods [49,50]. Use of a higher initial inoculum is expected to speed up the detection, and we estimate that with an inoculum of 10^6^ CFU·mL^−1^ the time to results could be lowered to 28 h. This would be similar to drug susceptibility testing using direct qPCR [51]. Finally, using additional preparation steps such as magnetic antibodies, it might be possible to skip the initial isolation, as a high amount of *Neisseria* would likely outgrow other microbes (if any) present after the purification step.

In conclusion, the use of isothermal microcalorimetry provides an interesting and useful tool to investigate fastidious microbes that require media incompatible with optical detection conventionally used in many commercial systems. Detection and drug susceptibility testing of *N. gonorrhoea* was rapidly performed. Further optimization with respect to the microbial culture condition will surely further decrease the time to detection and the time required for drug susceptibility testing. Therefore, we expect isothermal microcalorimetry to become a valuable tool for diagnostics and research of so-called fastidious microorganisms, including *N. gonorrhoea*.

## Figures and Tables

**Table 1:** Medium composition tested to measure growth parameters of *N. gonorrhoea* and define the best possible liquid medium.

No Supplement	Urine	Urine + Hemoglobin (1% *w/v*)	GC Medium	GC Medium + Hemoglobin (1% *w/v*)
Isovitale X	1% *v/v*	1% *v/v*	1% *v/v*	1% *v/v* *
Sheep blood **	1% *v/v*	1% *v/v*	1% *v/v*	1% *v/v*

* denotes the composition of the ATCC 814 medium. ** defibrinated.

**Table 2:** Growth parameters of *N. gonorrhoea* in the different media used. The results are the mean of four replicates and the associated standard deviation.

Growth Medium	Growth Rate (h^−1^)	Lag Phase (h)	Total Heat (J)	TTP (h)
GC	0.095 ± 0.010	39.4 ± 16.2	3.74 ± 1.30	67.5 ± 10.7
GC + Isovitale X	0.307 ± 0.015	28.8 ± 1.8	4.83 ± 0.38	40.8 ± 0.8
GC + Isovitale X + Blood	0.234 ± 0.025	19.6 ± 1.3	5.34 ± 0.14	39.6 ± 3.6
GC + hemoglobin	0.119 ± 0.016	16.7 ± 1.2	4.65 ± 0.17	36.2 ± 5.2
GC + hemoglobin + Isovitale X	***0.322 ± 0.025***	***17.3 ± 0.7***	***5.26 ± 0.12***	***29.4 ± 1.0***
GC + hemoglobin + Isovitale X + blood	0.305 ± 0.016	19.3 ± 1.0	5.47 ± 0.08	28.9 ± 2.4
Sterile GC medium	0.002 ± 0.001	ND	0.12 ± 0.05	ND
Urine	0.007 ± 0.001	ND	0.28 ± 0.08	ND
Urine + Isovitale X	0.012 ± 0.001	ND	0.59 ± 0.08	ND
Urine + Isovitale X + blood	0.011 ± 0.003	ND	0.47 ± 0.08	ND
Urine + hemoglobin	0.004 ± 0.003	ND	0.60 ± 0.71	ND
Urine + hemoglobin + Isovitale X	0.006 ± 0.003	ND	0.60 ± 0.51	ND
Urine + hemoglobin + Isovitale X + blood	0.004 ± 0.001	ND	0.36 ± 0.06	ND
Sterile filtered urine	0.004 ± 0.001	ND	0.62 ± 0.45	ND

ND: Not determined, TTP: Time to peak.

**Table 3:** Table showing the detection time with respect to the number of cells in the medium. The threshold for positive detection was set at 10 µW (10 µW is approximately 3.3 × 10^5^ CFU·mL^−1^; see main text).

ATCC 19424		ATCC 43069	
CFU·mL^−1^	Time to Detection (h)	CFU·mL^−1^	Time to Detection (h)
~2400	12.0 ± 0.3	~460	18.9 ± 0.4
~240	14.6 ± 0.2	~46	26.0 ± 0.4
~24	16.9 ± 0.4	~5	33.6 ± 3.3
~2	21.9 ± 3.2	~0.5 **	ND *
0	ND	0	ND
Sterile controls	ND	Sterile controls	ND

* Growth was detected, but the signal did not reach the threshold of 10 µW. ** At this concentration only one out of the three replicates showed growth. See figure for details.

## Data Availability

Data presented in this study are available on request from the corresponding author.
